# THE IMPACTS OF AGE AND PREPARATION INFORMATION ON PREPAREDNESS FOR PANDEMIC: A LATENT CLASS ANALYSIS

**DOI:** 10.1093/geroni/igad104.2674

**Published:** 2023-12-21

**Authors:** Zhirui Chen, Zhen Cong

**Affiliations:** The University of Alabama at Birmingham, Birmingham, Alabama, United States; The University of Alabama at Birmingham, Birmingham, Alabama, United States

## Abstract

This study examined the age differences in perceived and actual preparedness for a pandemic, as well as the moderating effect of preparation information. Data used was from 2021 FEMA National Household Survey (N = 6,305). Latent class analysis was first conducted to explore the typologies of the information respondents received about how to prepare for a pandemic based on eight indicators (e.g., basic survival, planning/preparation, testing, vaccines). With the adjustment of sample weights, four latent classes were identified: class 1 “insufficient information”, class 2 “information emphasizing disaster preparedness”, class 3 “information emphasizing disease preparedness”, and class 4 “comprehensive information”. Subsequently, the results of logistic regression and Poisson regression using sample weights showed that compared to those aged 60 and older, people aged 18-39 and aged 40-59 tended to perceive a lower level of preparedness but were more likely to engage in actual preparedness activities for a pandemic. Relative to those in “insufficient information” class, people in “information emphasizing disease preparedness” class and “comprehensive information” class were more likely to perceive a higher level of preparedness and take actual preparations for a pandemic. In terms of the interactions between age and preparation information, the gap narrowed between people aged over 60 and their younger counterparts in taking actual preparations if they were in “comprehensive information” class. These findings highlighted the age patterns in pandemic preparedness and the impacts of different preparation information strategies, which provided imperative implications for disaster preparedness education and communication campaigns.

